# Thioredoxin Reductase Is a Valid Target for Antimicrobial Therapeutic Development Against Gram-Positive Bacteria

**DOI:** 10.3389/fmicb.2021.663481

**Published:** 2021-04-16

**Authors:** LewisOscar Felix, Eleftherios Mylonakis, Beth Burgwyn Fuchs

**Affiliations:** Division of Infectious Diseases, Rhode Island Hospital, Alpert Medical School and Brown University, Providence, RI, United States

**Keywords:** allicin, antimicrobial, auranofin, drug resistance, ebselen, shikonin, thioredoxin system, thioredoxin reductase

## Abstract

There is a drought of new antibacterial compounds that exploit novel targets. Thioredoxin reductase (TrxR) from the Gram-positive bacterial antioxidant thioredoxin system has emerged from multiple screening efforts as a potential target for auranofin, ebselen, shikonin, and allicin. Auranofin serves as the most encouraging proof of concept drug, demonstrating TrxR inhibition can result in bactericidal effects and inhibit Gram-positive bacteria in both planktonic and biofilm states. Minimal inhibitory concentrations are on par or lower than gold standard medications, even among drug resistant isolates. Importantly, existing drug resistance mechanisms that challenge treatment of infections like *Staphylococcus aureus* do not confer resistance to TrxR targeting compounds. The observed inhibition by multiple compounds and inability to generate a bacterial genetic mutant demonstrate TrxR appears to play an essential role in Gram-positive bacteria. These findings suggest TrxR can be exploited further for drug development. Examining the interaction between TrxR and these proof of concept compounds illustrates that compounds representing a new antimicrobial class can be developed to directly interact and inhibit the validated target.

## Introduction

Years of antibiotic misuse and over prescription has taken a toll on the current drug arsenal, resulting in the emergence and expansion of drug resistant microbes. Even with improved hygiene practices and the implementation of antibiotic stewardship, the pharmacopeia is insufficient to remedy the evolving problem. There is a definitive need to develop new antimicrobial agents and define novel bacterial targets. Measures have been taken to engage in high throughput screens to identify previously unrecognized antimicrobial compounds and their respective targets (Moy et al., 2006; Hu et al., 2014; Katzianer et al., 2014; Rajamuthiah et al., 2014; Kim et al., 2015; Bageshwar et al., 2016; Ollinger et al., 2019). Here we pose that thioredoxin reductase (TrxR), part of the antioxidant thioredoxin system (TS), can serve as a new antimicrobial target in Gram-positive and a limited number of Gram-negative bacteria. Cells are constantly bombarded by reactive oxygen species, coming from environmental niches, hostile hosts in the form of immune responses, or even from the common task of metabolism and cellular respiration. Failure to maintain redox homeostasis leads to apoptosis.

Several compounds are recognized for inhibiting TrxR and provide proof of concept for validating this TrxR as an antimicrobial target. Within this review, we explore auranofin, shikonin, ebselen, and allicin as compounds that inhibit TrxR and remark on the antimicrobial activity profiles. The collective perspective is that TrxR can be targeted through new drug development with the aim of impacting predominately Gram-positive and glutathione independent bacteria.

## Thioredoxin System

**FIGURE 1:**
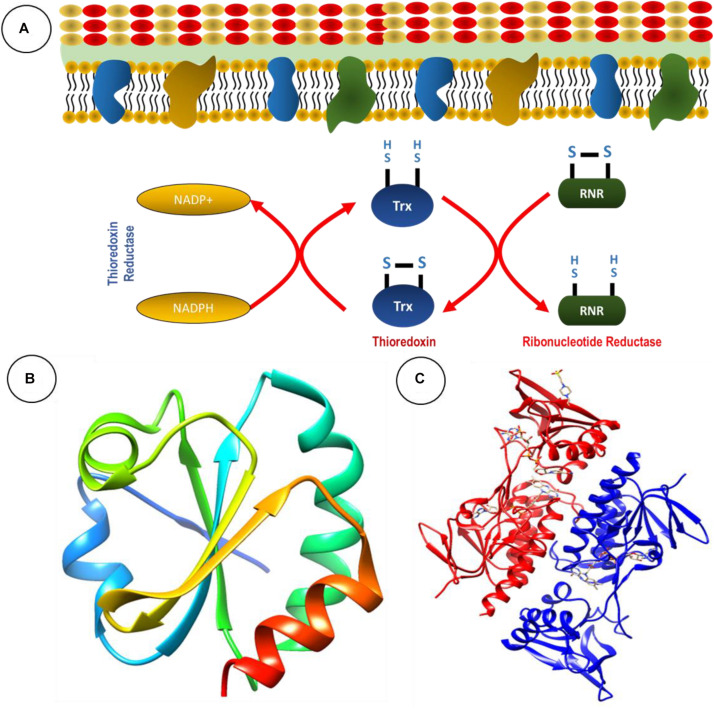
The thioredoxin system serves as an antioxidant system. **(A)** Basic chemical reaction of thioredoxin system in bacteria. Ribbon structure of **(B)** thioredoxin and **(C)** thioredoxin reductase from *Staphyloococcus aureus* generated through UCSF Chimera 1,14 software.

The TS is present in all living cells, inclusive of bacteria and fungi. Foremost, it serves as an antioxidant system through the conversion of thiol and disulfide bonds (Arnér and Holmgren, 2000). The TS is comprised of two antioxidant oxidoreductase proteins: thioredoxin (Trx) and TrxR, and an electron donor in the form of NADPH (Karlenius and Tonissen, 2010; Figure 1A; Table 1).

**TABLE 1:** Bacterial TS genes and their respective functions.

Bacteria	Trx Genes	TrxR Genes	Function	References
*Staphylococcus aureus*	*trxA*	*trxB*	Thioredoxin and thioredoxin reductase genes respond to oxygen and disulfide stress	Uziel et al., 2004
*Helicobacter pylori*	*trxA* and *trxC*	*trxR*	Required for colonization and survival	Baker et al., 2001
*Mycobacterium tuberculosis*	*trxA, trxB* and *trxC*	*trxR*	Needed for survival against reactive oxygen and nitrogen species produced by activated macrophages	Trivedi et al., 2012
*Bacillus subtilis*	*TrxA*	*trxA* and *trxB*	Needed for survival and virulence	Zheng et al., 2019
*Escherichia coli*	*trxA* and *trxC*	*trxB*	Used to prevent oxidative damage from reactive nitrogen intermediates	St. John et al., 2001; Potamitou et al., 2002

Thioredoxin is a 10- to 12-kDa ubiquitous protein with a conserved catalytic site (Cys-Pro-Gly-Cys) (Lu and Holmgren, 2014). The primary role of Trx is to serve as a disulfide oxidoreductase and interact with a broad range of proteins involved in electron transport during substrate reduction or regulate the activity by controlling thiol-redox (Lu and Holmgren, 2014). Trx exerts influence of the overall redox system by reducing ribonucleotide reductase during DNA and protein repair and regulating transcription factors, suggesting significant involvement in cellular redox homeostasis (Lu and Holmgren, 2014). The ribbon structures of thioredoxin and TrxR from *Staphylococcus aureus* are given in Figures 1B,C.

Thioredoxin reductase is part of the pyridine nucleotide-disulfide oxidoreductase family. The average subunit mass in bacteria, archaea, and lower eukaryotes is approximately 35 kDa (Williams et al., 2000). Some of the familiar examples are mercuric ion reductase, lipoamide dehydrogenase, glutathione reductase, and NADH oxidase (Lu and Holmgren, 2014). Prokaryotic TrxR have high substrate specificity (Becker et al., 2000; Gromer et al., 2004), while NADPH and FAD are the common binding sites present in TrxR of bacteria (Gromer et al., 2004). In the process of TrxR catalysis, NADPH reduces the FAD of TrxR, and then FAD reduces Trx by disulfide exchange. This mechanism provides reducing equivalents to other target proteins essential for maintaining cellular redox homeostasis (Becker et al., 2000).

Thioredoxin reductase contains the rare amino acid selenocysteine (Sec), making it a selenoprotein. Sec is a cysteine analog in which selenium replaces the sulfur present in the cysteine. It is a rare naturally occurring amino acid, known to be in 54 human proteins (Parsonage et al., 2016). The sequence UGA normally act as a stop codon; however, when the UGA codon is followed by an Sec insertion sequence (SecIS) in bacteria Sec is added through co-translational insertion (Parsonage et al., 2016).

Thioredoxin reductase importance in maintaining systemic redox homeostasis has been noted not only in Gram-positive bacteria but several parasites as well, where researchers have shown interruption of the enzyme presents a potential therapeutic target (Bonilla et al., 2008; Angelucci et al., 2009; Debnath et al., 2013; Andrade and Reed, 2015; Parsonage et al., 2016). Parsonage and colleagues examined TrxR structure from the parasite *Entamoeba histolytica* using crystallography and found auranofin interaction with Cys286 (Parsonage et al., 2016). However, a Cys286 TrxR mutant retained catalytic activity and auranofin susceptibility leading to the suggestion that it is a decoy site and binding is not fully dependent on this interaction. The authors also suggest that auranofin interaction alone with TrxR may not be sufficient to inhibit the *E*. *histolytica*, indicating the potential for additional targets. Angelucci et al. (2009) present alternative findings in the parasite *Schistosoma mansoni* where a glutaredoxin domain is fused with a TrxR domain to create thioredoxin-glutathione reductase. It is indicated by the data of this study, that auranofin interacts with thioredoxin-glutathione reductase through a selenocysteine mediated transfer of gold from auranofin to Cys sites and gold provides the inhibitory activity, essentially making auranofin the pro-drug for gold delivery (Angelucci et al., 2009). It is not yet be fully elucidated how auranofin interaction with TrxR in Gram-positive bacteria leads to inhibition but there is the potential that the CXXC motif could be a target. Another parasite, *Leishmania infantum*, offers insight into auranofin interference of the redox system by inhibiting trypanothione reductase, an enzyme that replaces many of the thioredoxin/TrxR and glutathione/glutathione reductase functions. Looking at the X-ray crystal structure of auranofin-trypanothione reductase-NADPH complex, gold was found bound to two cysteine residues (Cys52 and Cys57) while the sugar moiety bound to the docking site from another parasite, *L. infantum* (Ilari et al., 2012).

Where we have seen potential to target TrxR in Gram-positive bacteria and the functional orthologs in parasites, this opportunity is lacking for Gram-negative bacteria. In most Gram-negative bacteria, the TS is backed up by a glutathione-glutaredoxin (GSH) system that is also able to scavenge reactive oxygen and nitrogen species in order to maintain redox homeostasis (Couto et al., 2016). Therefore, TrxR lacks the essential nature in Gram-negative bacteria that it plays in Gram-positive bacteria. The GSH system is comprised of NADPH, glutathione reductase, glutathione, and glutaredoxin (Lu and Holmgren, 2014). The Gram-negative *Helicobacter pylori* proves to be an exception to the classification rule as a GSH-independent bacterium (Lu and Holmgren, 2014). Further, *Mycobacterium tuberculosis*, which does not fit within the Gram classification, is TrxR-dependent and GSH-independent (Lu and Holmgren, 2014). This suggests that compounds directly inhibiting the TS, especially through TrxR, hold the potential to inhibit medically important Gram-positive bacteria such as *Staphylococcus* spp., *Streptococcus* spp., *Enterococcus* spp., or the exceptional GSH independent bacteria *H*. *pylori* and *M*. *tuberculosis*.

## Reliance on Trx and TrxR in GSH-Independent Bacteria

*Helicobacter pylori* is an interesting organism for TS studies as one of the only Gram-negative bacteria to be GSH-independent. The important antioxidants TS system in this bacterium is comprised of one TrxR and two Trx (Trx1 and Trx2) proteins (Table 1; Windle et al., 2000). Though most bacteria fit within the Gram classification system, *M. tuberculosis* is another bacterium that does not, and also represents a GSH-independent bacterium utilizing the TS. It has three Trx proteins, TrxA, TrxB, and TrxC, but only one TrxR (Table 1). However, *M. tuberculosis* TrxR does not transfer electrons to TrxA, making TrxB and TrxC the recipients and the active disulfide reductases (Trivedi et al., 2012). *M. tuberculosis* also has thiol peroxidase (Tpx) and alkyl hydroperoxide peroxidase (AhpC) that respond to oxidative and nitrosative stresses imposed by macrophages. Similarly, *H*. *pylori* also utilizes AhpC as an essential antioxidant for maintaining redox hemostasis (Baker et al., 2001). In *M*. *tuberculosis*, TrxB transfers electrons to Tpx, and TrxC can transfer to either Tpx or AhpC (Lu and Holmgren, 2014). As GSH-independent bacteria, *H*. *pylori* and *M*. *tuberculosis* are susceptible to TrxR targeting compounds, both demonstrating sensitivity to auranofin (Harbut et al., 2015; Owings et al., 2016). Thus, GSH designation as dependent or independent has thus far proven to influence bacterial susceptibility to TrxR targeting compounds and presents a new bacterial classification outside of the well-defined Gram system.

## TrxR Inhibitors and the Impact to Microbes

**FIGURE 2:**
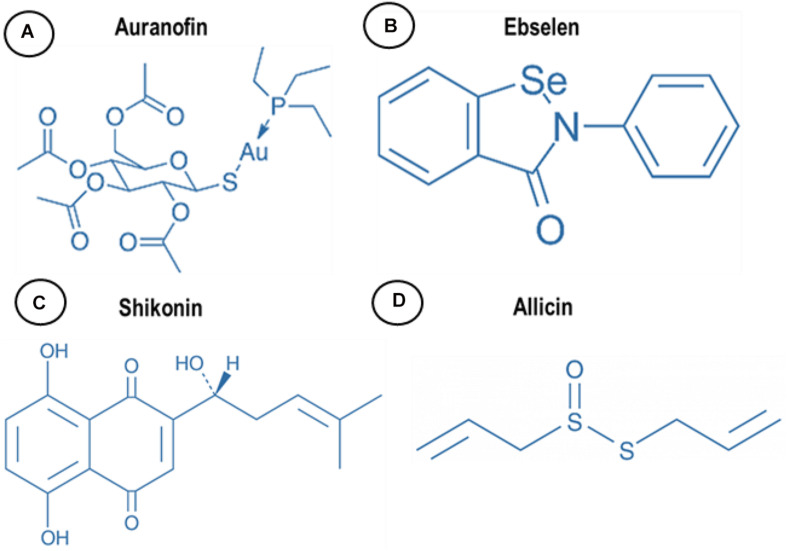
Chemical structure of **(A)** auranofin, **(B)** ebselen, **(C)** shikonin, and **(D)** allicin.

There are several drugs and molecules that target TrxR. Two naturally occurring compounds, shikonin and allicin (Figure 2), are produced by plant roots, presumptively to combat bacteria in the rhizosphere, an indication that nature has already identified TrxR as an antimicrobial target site. Screening efforts to explore the pharmacopeia for antimicrobial compounds also identified auranofin and ebselen for antimicrobial properties that appear to also target TrxR (Figure 2). By understanding and repurposing these molecules, we can define a path toward building a new class of antimicrobial compounds that specifically target bacterial TrxR.

Among the four molecules, auranofin has the lowest minimal inhibitory concentration (MIC) against most of the Gram-positive bacteria. Auranofin has shown efficacy toward *S. aureus* (0.0625–1 μg/mL), *Enteroccocus faecalis* (0.125–0.5 μg/mL), *Enterococcus faecium* (0.125–0.25 μg/mL), *Clostridium difficile* (0.25–4 μg/mL), *Bacillus subtilis* (0.3–0.05 μg/mL), *Streptococcus pneumoniae* (0.025–0.25 μg/mL), and *Streptococcus agalactiae* (0.0625–0.0015 μg/mL) (Harbut et al., 2015; Thangamani et al., 2016a; AbdelKhalek et al., 2019). The MIC of shikonin against Gram-positive bacteria including drug resistant methicillin-resistant *S. aureus* (MRSA) ranged between 6.5 and 31.2 μg/mL, thus showing a propensity to inhibit bacteria but not with the same impact as auranofin (Vegara et al., 2011; Lee et al., 2015).

Ebselen exhibited efficacy at inhibiting Gram-positive bacteria including: *B. subtilis*, *Bacillus cereus*, *S. aureus* [MRSA, Linezolid-resistant *S. aureus*, Mupirocin-resistant *S. aureus*, vancomycin resistant *S. aureus* (VRSA), and vancomycin intermediate *S. aureus* (VISA)] (MIC 0.125–0.64 μg/mL), *Staphylococcus epidermidis* (MIC 0.5 μg/mL), *E. faecium* (MIC 0.25–4 μg/mL), *E. faecalis* (MIC 0.125–2 μg/mL), *Streptococcus pyogenes* (MIC 0.5 μg/mL), and *S. agalactiae* (MIC 0.5 μg/mL) (Thangamani et al., 2015a, b; Gustafsson et al., 2016; AbdelKhalek et al., 2019). Allicin is the least potent among the group with activity against Gram-positive bacteria like *S. aureus* at an MIC of 32–64 μg/mL (Leng et al., 2011).

## Auranofin

Auranofin is an FDA approved gold containing compound used to treatment rheumatoid arthritis. Since auranofin has been used since the mid 1980s and was even provided in studies to children ages 1–17, information is available related to drug safety at therapeutic dosages pertinent to arthritis (Kean et al., 1997). When provided orally at 6 mg/day, 25% of the administered compound is detected in the plasma bound to albumin with a half-life of 15–25 days. Approximately 55–80 days is required to fully eliminate the drug with excretion routes being urine and feces. Bioavailability is 40 and 60% protein binding (Finkelstein et al., 1976; Kean et al., 1997). The most common side effect encountered when using auranofin to treat arthritis was diarrhea and skin rash (Felson et al., 1990). Diarrhea was experienced by 40% of patients and rash occurred in 20% of patients (Kean et al., 1997). Proteinuria was also reported in 5% of treated patients and is hypothesized to be a result of gold or gold complexes from auranofin damaging the renal tubule. Auranofin manifest reduction in arthritis stiff joints, reduces the number of swollen joints, and improves grip strength through eliciting a reduction in blood IgG, decreasing α2-globulin, increasing albumin ratios, and decreasing rheumatoid factor titers (Finkelstein et al., 1976).

## Auranofin Bacterial Inhibitory Mechanism

A high throughput screen identified auranofin for prolonging the survival of the invertebrate model *Caenorhabditis elegans* infected with *S*. *aureus*. The results demonstrated that auranofin could clear bacteria from the host at low concentrations (0.78 mg/mL), but the inhibitory impact was reduced by secondary oxidative scavenger glutathione (Fuchs et al., 2016). Indeed, auranofin has been identified by other screening efforts. In 2016, auranofin emerged as a hit when searching for compounds that disrupt preformed *S. aureus* biofilm (Torres et al., 2016).

The *S*. *aureus* target for auranofin was confirmed when it was shown to provide dose dependent inhibition of bacterial TrxR. Enzymatic activity was evaluated through a colorimetric assay where DTNB is reduced with NADPH to become TNB in an assay catalyzed by TrxR. The reaction produces a yellow color but the color is reduced by the presence of a TrxR inhibitor (Tharmalingam et al., 2019).

This was not the first implication that auranofin could inhibit TrxR. Using the colorimetric assay where DTNB is reduced with NADPH to become TNB in the presence of TrxR, Harbut and colleagues showed auranofin appears to directly reduce the enzymatic activity of *M*. *tuberculosis* and *S*. *aureus* TrxR in a dose-dependent manner (Harbut et al., 2015). Lin et al. (2016) used a conditional mutant to show TrxR targeting reduced bacterial burden in a mouse infection model, concluding that auranofin targets TrxB2 but acknowledge this may not be the only enzyme affected.

There are possibly other targets or influences asserted by auranofin. Thangamani et al. (2016a) suggested that auranofin has antibacterial activity against Gram-positive bacteria by inhibiting several biosynthesis mechanisms or pathways like protein, DNA, and cell wall synthesis. The authors observed suppressed protein synthesis in *S. aureus* leading to reduced MRSA toxin productivity.

## Auranofin Inhibits Bacterial Pathogens

Auranofin is able to inhibit a significant cache of bacteria and exhibited antibacterial activity against non-replicating and replicating strains of *M. tuberculosis* under nutrient deprivation. After 5 days, 1.3 and 3.7 log reductions in bacterial viability were recorded when treated with 100 nM and 1.0 μM of auranofin, respectively (Harbut et al., 2015). The MIC of auranofin against *M. tuberculosis* (Δ*panCD*,Δ*RD1*) and *M. tuberculosis* H37Ra was 0.5 μg/mL, while the MIC of *B. subtilis* was between 0.3 and 0.05 μg/mL (Harbut et al., 2015). Clinical isolates of *C. difficile* were inhibited up to 50% by 1 μg/mL of auranofin. At this concentration, the toxin productivity and spore formation of *C. difficile* were also inhibited. Clinical and reference strains of *C. difficile* exhibited an MIC between 0.25 and 4 μg/mL. Reference strains of *E. faecalis* and *E. faecium* had an MICs ranging between 0.125–1 and 0.25–4 μg/mL, respectively (Harbut et al., 2015; Thangamani et al., 2016a; AbdelKhalek et al., 2019).

*Staphylococcus aureus* has demonstrated susceptible to auranofin, inclusive of drug resistant isolates. Reference strains, clinical isolates, MRSA, VISA, VRSA, linezolid resistant *S*. *aureus*, and glycopeptide intermediate *S. aureus* exhibited MICs ranging between 0.0625 and 1 μg/mL (Harbut et al., 2015; Thangamani et al., 2016a; AbdelKhalek et al., 2019). In 2019, auranofin was tested to have an MIC ranging between 0.125 and 1 μg/mL against 503 clinical isolates of *S*. *aureus* (Tharmalingam et al., 2019). *S. agalactiae* also demonstrated susceptibility with MICs ranging between 0.0625 and 0.0015 μg/mL. Thus, showing susceptibility of Gram-positive bacteria and lack of resistance, even among drug resistant isolates.

## Antimicrobial Efficacy in Animal Models

Auranofin antimicrobial efficacy extends beyond *in vitro* data and was found to have an *in vivo* impact by prolonging survival of mice infected with Gram-positive bacteria in several reports (Table 2). Provision of auranofin in systemic, peritonitis, and topical infection mouse models inhibited MRSA, demonstrated through bacterial load reduction (Thangamani et al., 2016b).

**TABLE 2:** *In vivo* efficacy of auranofin in animal models.

Mouse models	Route of administration	Bacterial strains	Result of the experiment	References
Skin infection model	Topical (petroleum gel as vehicle)	*S*. *aureus* (MRSA USA300)	Reduction of MRSA CFU (3.64 ± 0.14 log_10_) Reduction in inflammatory cytokines tested (IL-6, IL-1β, TNFα and MCP-1)	Thangamani et al., 2016b
Murine systemic infection	Peritoneal	*S*. *aureus* (MRSA strain Sanger 252)	Survival of mice after for 7 d after treatment	Harbut et al., 2015
Peritonitis–sepsis infection model	Oral	*S*. *pneumoniae* serotype 23F strain; serotype 8 strain (strain 3498)	Reduced mortality and 50% of mice survived Reduction in bacterial load within 24h	Aguinagalde et al., 2015
Intramuscular infection	Subcutaneous	*S*. *aureus* (MRSA 132)	Reduction of bacterial load within 24h	Aguinagalde et al., 2015
Mesh associated biofilm infection	Intraperitoneal	*S*. *aureus* (MRSA 132)	Decrease in bacterial load attached to the mesh	Aguinagalde et al., 2015
*C*. *difficile* infection model	Intraperitoneal	*C. difficile*	Protected 100% of mice at lowest concentration of drug (0.25 mg/Kg) from *C*. *difficile* infection	Abutaleb and Seleem, 2020
Infected pressure ulcer wound model in obese mice	Topical	*S. aureus* MRSA	8-log_10_ reduction in MRSA	Mohammad et al., 2020

Oral administration of auranofin (0.125 or 0.25 mg/kg) promoted survival in 80% of mice post MRSA infection in a lethal septicemic mouse model. Similar survival rates (80%) were observed in mice treated with linezolid (25 mg/kg) showing comparable results with a standard of care medication. Further, auranofin reduced mean bacterial load by 90% in the liver compared to a 70% reduction in animals treated with linezolid. Auranofin given to mice infected with a non-lethal dose of MRSA USA300 reduced the bacterial load up to 95% in the spleen (Thangamani et al., 2016b). In another infection model, Harbut et al. (2015) tested auranofin in CD1 female mice using a MRSA peritonitis model. The animals were injected with 0.12 or 0.012 mg/kg of auranofin daily. After treatment, four out of eight mice (50%) experienced prolonged survival by day 7 when provided with 0.12 mg/kg auranofin compared to three out of eight mice (37.5%) survived to day 7 of the study when provided with 0.012 mg/kg (Harbut et al., 2015).

The *in vivo* efficacy of auranofin was also tested against the drug resistant strain *S. pneumoniae* 48 (serotype) in a mouse bacteremia model. After auranofin treatment at the concentrations of 1, 5, or 10 mg/kg mouse survival was observed at 50, 30, and 20%, respectively within a 96-h period. The same time interval results in 100% mortality of infected mice in the placebo group. Dosing with 1 mg/kg auranofin was determined to significantly prolong survival and no trace of bacteria was reported in tail vein collected blood samples 24 h after of infection (Aguinagalde et al., 2015). The greater efficacy at lower doses of 1 and 5 mg/kg suggested in this study by Aguinagalde et al. (2015) implicate potential influence by the paradoxical Eagle effect as seen with other antibiotics, a phenomenon where a decrease in net cell death occurs when drug concentrations exceed optimal bactericidal levels (Eagle, 1948; Prasetyoputro et al., 2019).

The same study interrogated MRSA strain 132 susceptibility to auranofin in an intramuscular infection model. At 24 h post infection, bacteria were enumerated revealing a significant reduction when provided with 5 mg/kg auranofin compared to untreated mice (Aguinagalde et al., 2015). Using the same 5 mg/kg auranofin concentration, it was also reported to reduce MRSA biofilm seeded onto mesh implanted intraperitoneally. After 6 days, auranofin daily therapy significant reduced the number of *S*. *aureus* CFUs recovered from the recovered mesh compared to untreated mice (Aguinagalde et al., 2015).

## Broad Scope Efficacy

There is evidence that auranofin impact on infectious diseases extends beyond Gram-positive bacteria and includes parasites. Auranofin inhibits *E. histolytica*, a parasite that causes diarrhea, by targeting TrxR (Andrade and Reed, 2015; Parsonage et al., 2016). It also affects *Giardia lamblia* and *S. mansoni* (Andrade and Reed, 2015). It is from these parasitic interactions that we gain valuable insight into the physical interaction of auranofin with the TrxR target. The ability to inhibit both bacteria and parasites lend to the potential of elevating auranofin or other effective TrxR targeting compounds to become broad-spectrum anti-infectious therapeutic agents.

## Ebselen

Like auranofin, the antimicrobial activity for ebselen was also revealed in a high throughput screening effort to find compounds that inhibit *S*. *aureus* and was found to have greater impact on planktonic cells than biofilm (Torres et al., 2016). Ebselen, chemically known as 2-phenyl-1,2-benzisoselenazol-3(2H)-one, is a seleno-organic, non-toxic drug reported to have antiatherosclerotic, anti-inflammatory, and cytoprotective properties (Schewe, 1995). It was initially developed to mimic the structure of glutathione peroxidase (Müller et al., 1984), but also reacts with peroxynitrite and can inhibit enzymes such as lipoxygenases, NO synthases, NADPH oxidase, protein kinase C, and H^+^/K^+^-ATPase (Parnham and Sies, 2000). In mammalian cells, ebselen is a favorable substrate for TrxR dependent detoxification of hydroperoxides (Müller et al., 1984).

Under *in vitro* analysis, ebselen exhibited antibacterial activity against MRSA strains USA300, USA100, USA200, USA500, USA1000, USA1100, linezolid resistant, VRSA and mupirocin resistant strains of *S. aureus* (King et al., 2006; Thangamani et al., 2015a), helping to bolster the idea that there is *S*. *aureus* broad scope susceptibility regardless of strain or drug resistance characteristics. Indeed, the inhibitory activity against *S*. *aureus* was robust with an MIC at 0.5 mg/mL (Thangamani et al., 2015a). The observed antimicrobial activity extends to other Gram-positive bacteria such as *Streptococcus* and *Enterococcus*. Once again, *H*. *pylori* proved to be an exceptional Gram-negative bacteria, exhibiting sensitivity to ebselen with an MBC at 3.13–12.5 mg/mL and an MIC at 3.13 mg/mL for various isolates (Lu et al., 2013). Additionally, *M*. *tuberculosis* demonstrated ebselen sensitivity with an MIC at 20 μg/mL, congruent to findings with auranofin (Lu et al., 2013). Susceptible bacteria did not easily develop resistance to ebselen as demonstrated by Gustafsson et al. (2016) who were unable to induce *S*. *aureus* or *B*. *subtilis* resistance after extended exposure. This could be due to the essential nature of the currently identified TrxR target or suggest the compound has a larger impact and affects other cellular systems.

By measuring Trx reduction in a colorimetric assay with DTNB, Lu et al. (2013) demonstrate that ebselen acts as a competitive inhibitor to *E*. *coli* TrxR. When ebselen was removed from the reaction through membrane filtration, 80% of TrxR activity was restored (Lu et al., 2013). However, unlike auranofin, which was effective at reducing bacterial growth and lysed *M*. *tuberculosis*, ebselen did not work via TrxB inhibition and likely affects alternative targets in this bacterium (Lin et al., 2016).

## Shikonin

The napthoquinone shikonin is a Chinese herbal medicine, also known as Zicao, isolated from *Lithospermum erythrorhizon* roots, a plant known for wound healing and anti-inflammatory effects (Andujar et al., 2013; Duan et al., 2014; Lee et al., 2015). There are several Gram-positive bacteria with demonstrated sensitivity to shikonin: *S. aureus*, *E. faecium*, and *B. subtilis*, with MICs ranging from 0.3 to 6.25 mg/mL (Andujar et al., 2013). Shikonin also prevents *H*. *pylori* growth in a dose dependent manner, reaching 86% inhibition at 40 μM (Kuo et al., 2004).

Duan and colleagues report that shikonin is yet another compound that interacts with cytosolic TrxR. The compound targets the Sec residue of the Gly-Cys-Sec-Gly active site motif of the human TrxR and irreversibly inhibits the enzyme (Duan et al., 2014). This interaction inhibits TrxR activity without depleting the cellular accumulation of the enzyme (Duan et al., 2014). Shikonin activity can be inhibited by the inclusion of N-acetyl-L-cysteine, a known antioxidant and a precursor to GSH, which neutralizes ROS as part of the alternate glutathione synthase pathway, or through the over expression of TrxR itself (Duan et al., 2014). Thus, shikonin appears to target the TS antioxidant activity as asserted by antagonistic effect when alternate cellular antioxidants are included. Although shikonin inhibits *S*. *aureus*, it does not reach the same impact as auranofin which exhibited lower MICs. The lesser inhibitory activity is likely due to impeded cellular entry due to factors that restrict shikonin passive entry. It is likely impeded by the cell membrane but inclusion of membrane permeabilization agents such as Triton X-100 enhances antimicrobial activity against *S*. *aureus* (Lee et al., 2015). Entry challenges are affirmed when peptidoglycans is added and shows competitive inhibition (Lee et al., 2015).

Although it has been used in an animal models, there are distinct challenges that impede shikonin moving forward as a widely antimicrobial therapeutic agent such as poor solubility. Also, shikonin can elicit adverse toxic effects. Although shikonin presents challenges of advancing as an antimicrobial agent in a clinical setting, it does highlight TrxR as a viable antibacterial target and present scaffolding structure that can be built upon for improved activity. And, importantly, it provides an example of a natural product that targets TrxR.

## Allicin

Another natural plant derived compound that exerts antimicrobial effects is allicin, a biologically active oxygenated sulfur compound present in freshly crushed garlic extract that is chemically known as thio-2-propene-1-sulfinic acid *S*-allyl ester (Ankri and Mirelman, 1999). In 1944, Cavallito and Bailey (1944), first identified allicin and reported the antibacterial activity of diluted allicin against Gram-positive and Gram-negative bacteria. Later, in 1970, the antifungal activity of allicin was revealed (Feldberg et al., 1988). It was further identified to have antiparasitic activity against *E. histolytica* and *G. lamblia* (Ankri and Mirelman, 1999).

Allicin exerts antibacterial activity against *S. aureus* in combination or synergistically with cefazolin/oxacillin and cefoperazone (Cai et al., 2007). When allicin is provided at 100 μg/mL it prevents *S. aureus* biofilm formation on implant materials like reticular polypropylene meshes used in hernia repair (Pérez-Köhler et al., 2015a, b). When biofilm inhibitory activity was tested in an *in vivo* rabbit model, allicin (delivered at 4 mg/L) significantly inhibited the formation of *S. epidermidis* biofilm formation (Zhai et al., 2014).

Like auranofin, allicin is also able to inhibit *H*. *pylori*, possibly due to the GSH-independent status of the microbes (Jonkers et al., 1999; Aydin et al., 2000; Koçkar et al., 2001). The MIC and MBC were both measured at 6 μg/mL against *H*. *pylori* (O’Gara et al., 2000). In a cohort consisting of 210 *H*. *pylori* positive patients, a group (*n* = 30) treated with standard care medications lansaprasol, clarithromycin, and amoxicillin experienced eradication in 66.6% of the group. When allicin was added to the treatment regimen at 4200 μg/day eradication reached 90% within the group (Koçkar et al., 2001).

*Mycobacterium tuberculosis* was also sensitive to allicin, exhibiting MICs ranging from 2.58 to 33.33 μg/mL among drug resistant and drug susceptible isolates (Dwivedi et al., 2019). With multiple types of microbes inhibited by allicin, it is not surprising that its mode action is promiscuous. Allicin undergoes a chemical reaction with thiol groups present in several enzymes including TrxR, RNA polymerase, and alcohol dehydrogenase (Ankri and Mirelman, 1999). Muller et al. (2016) measured total sulfhydryl concentrations and demonstrated a reduction using crude extracts from cells treated with allicin. Sulfhydryl levels were quantified with DTNB (described above) and showed that TrxR is one of the targeted enzymes.

## Cidal and Static Activity

Natural and synthetic drugs exhibit two types of inhibitory effects on bacteria: bacteriostatic and bactericidal. The MIC is the concentration at which a compound inhibits the visible growth of bacterial at 24 h under optimum condition. While the minimum bactericidal concentration (MBC) is defined as the point at which a compound causes 1,000 fold bacterial density reduction at 24 h under standardized growth conditions (Wald-Dickler et al., 2018). Bactericidal activity is defined when the ratio of MBC to MIC is ≤4 and bacteriostatic activity is defined as the MBC to MIC ratio is >4. Therefore, a drug which achieves reduction of >1,000 fold in density of bacteria at concentration eight-fold above the MIC is considered bacteriostatic (Pankey and Sabath, 2004; French, 2006; Nemeth et al., 2015; Wald-Dickler et al., 2018). Thus, effective bactericidal compound needs to have an MBC close to the MIC.

Auranofin has been reported to exhibit bactericidal activity against Gram-positive bacteria like *B. subtilis*, *C. difficile*, *E. faecalis*, *E. faecium*, *S. aureus* (including MRSA and VISA strains), *S. epidermidis, S. pneumoniae*, and *S. agalactiae*. Reports also suggest bactericidal activity of auranofin against *M. tuberculosis* (Harbut et al., 2015; Thangamani et al., 2016b; AbdelKhalek et al., 2019). The MBC to MIC ratio of shikonin is reported above >4 against various strains of *S. aureus*. This suggest that shikonin exhibits bacteriostatic activity (Vegara et al., 2011; Lee et al., 2015). Ebselen shows MBC to MIC ratio is <4 against Gram-positive bacteria like *B. subtilis*, *Enterococcus* spp. (including VRE), *S. epidermidis*, *S. aureus* (including MRSA, VRSA, and VISA strains), *S. pyogenes*, and *S. agalactiae* (Thangamani et al., 2015b; Gustafsson et al., 2016; AbdelKhalek et al., 2018). Apart from these three molecules, allicin is reported to inhibit *S*. *aureus* at >32 μg/mL. This suggest that allicin possess bacteriostatic activity. Thus, among these four molecules (auranofin, shikonin, ebselen, and allicin), auranofin is potentially the most active at inhibiting bacteria in a bactericidal capacity.

## Conclusion

Although there are differences between auranofin, ebselen, shikonin, and allicin, together, these compounds strongly suggest TrxR is a valid antimicrobial target. Although all of the compounds commonly impact TrxR there is debate as to whether it is the sole target. Indeed, other cellular systems may be augmented.

Only auranofin has been used clinically (with FDA approval) and found to be non-toxic. Therefore, holding the greatest potential to be repositioned for antimicrobial therapy. Interestingly, studies suggest TrxR inhibitors could also have anti-cancer efficacy due to increased growth rates and elevated cellular ROS from increased mitochondrial respiration that accumulate during accelerated growth resulting in high levels of Trx and TrxR to reduce oxidative stressors (reviewed in Mohammadi et al., 2019). Clinical trials are ongoing using a TrxR inhibitor ethaselen to treat non-small cell lung cancers.

Focusing on the common target, each of these compounds have been shown to reduce TrxR enzymatic activity and the inhibition of bacterial growth has been demonstrated with *in vitro* and *in vivo* assays. Antimicrobial impact is best reflected in Gram-positive bacteria with the exception of a couple of GSH-independent microbes, namely *H*. *pylori* and *M*. *tuberculosis*. Multiple studies demonstrate that drug resistant microbes are susceptible to TrxR targeting compounds and resistance is not developed upon exposure. Among GSH-independent bacteria, the essential nature of TrxR makes it a very attractive target. Although this review is restricted to bacterial inhibition, TrxR also poses a potential target in fungi. Indeed, Missall and Lodge (2005) and Ianiri and Idnrum (2015) report the essential nature of fungal TrxR in *Cryptococcus neoformans* with failure to produce a mutant strain.

The quickest path forward to introducing TrxR as a novel antimicrobial target not yet tapped by current antibiotics is to progress one of the existing compounds as a repurposed drug. With low toxicity and good efficacy at low concentrations, the best candidate for this ascension is auranofin. However, the creation of new compounds specifically designed to target Gram-positive TrxR can initiate a new antimicrobial class.

## Author Contributions

BF and LF prepared the initial draft. EM provided insightful discussions and contributed to the writing. All authors contributed to the article and approved the submitted version.

## Conflict of Interest

The authors declare that the research was conducted in the absence of any commercial or financial relationships that could be construed as a potential conflict of interest.

## References

[B1] AbdelKhalek A. Abutaleb N. S. Mohammad H. Seleem M. N. (2018). Repurposing ebselen for decolonization of vancomycin-resistant enterococci (VRE). *PLoS One* 13:e0199710. 10.1371/journal.pone.0199710 29953486 PMC6023106

[B2] AbdelKhalek A. Abutaleb N. S. Mohammad H. Seleem M. N. (2019). Antibacterial and antivirulence activities of auranofin against *Clostridium* difficile. *Int. J. Antimicrob. Agents* 53 54–62. 10.1016/j.ijantimicag.2018.09.018 30273668 PMC6475173

[B3] Abutaleb N. S. Seleem M. N. (2020). Auranofin, at clinically achievable dose, protects mice and prevents recurrence from *Clostridioides* difficile infection. *Sci. Rep.* 10:7701. 10.1038/s41598-020-64882-9 32382070 PMC7206065

[B4] Aguinagalde L. Díez-Martínez R. Yuste J. Royo I. Gil C. Lasa Í, et al. (2015). Auranofin efficacy against MDR Streptococcus pneumoniae and *Staphylococcus aureus* infections. *J. Antimicrob. Chemother.* 70 2608–2617. 10.1093/jac/dkv163 26142477

[B5] Andrade R. M. Reed S. L. (2015). New drug target in protozoan parasites: the tole of thioredoxin reductase. *Front. Microbiol.* 6:975. 10.3389/fmicb.2015.00975 26483758 PMC4588103

[B6] Andujar I. Recio M. Giner R. Ríos J. (2013). Traditional chinese medicine remedy to jury: the pharmacological basis for the use of shikonin as an anticancer therapy. *Curr. Med. Chem.* 20 2892–2898. 10.2174/09298673113209990008 23651309

[B7] Angelucci F. Sayed A. A. Williams D. L. Boumis G. Brunori M. Dimastrogiovanni D. (2009). Inhibition of *Schistosoma mansoni* thioredoxin-glutathione reductase by auranofin: structural and kinetic aspects. *J. Biol. Chem.* 284 28977–28985. 10.1074/jbc.M109.020701 19710012 PMC2781444

[B8] Ankri S. Mirelman D. (1999). Antimicrobial properties of allicin from garlic. *Microbes Infect.* 1 125–129. 10594976 10.1016/s1286-4579(99)80003-3

[B9] Arnér E. S. J. Holmgren A. (2000). Physiological functions of thioredoxin and thioredoxin reductase. *Eur. J. Biochem.* 267 6102–6109. 10.1046/j.1432-1327.2000.01701.x 11012661

[B10] Aydin A. Ersöz G. Tekesin O. Akçiçek E. Tuncyürek M. (2000). Garlic oil and *Helicobacter pylori* infection. *Am. J. Gastroenterol.* 95 563–564. 10.1016/S0002-9270(99)00871-0 10685782

[B11] Bageshwar U. VerPlank L. Baker D. Dong W. Hamsanathan S. Whitaker N. (2016). High throughput scree for *Escherichia coli* twin arginine translocation (Tat) inhibitors. *PLoS One* 11:e0149659. 10.1371/journal.pone.0149659 26901445 PMC4764201

[B12] Baker L. M. S. Raudonikiene A. Hoffman P. S. Poole L. B. (2001). Essential thioredoxin-dependent peroxiredoxin system from *Helicobacter pylori*: genetic and kinetic characterization. *J. Bacteriol.* 183 1961–1973. 10.1128/JB.183.6.1961-1973.2001 11222594 PMC95091

[B13] Becker K. Gromer S. Heiner Schirmer R. Müller S. (2000). Thioredoxin reductase as a pathophysiological factor and drug target. *Eur. J. Biochem.* 267 6118–6125. 10.1046/j.1432-1327.2000.01703.x 11012663

[B14] Bonilla M. Denicola A. Novoselov S. V. Turanov A. A. Protasio A. Izmendi D. (2008). Platyhelminth mitochondrial and cytosolic redox homeostasis is controlled by a single thioredoxin glutathione reductase and dependent on selenium and glutathione. *J. Biol. Chem.* 283 17898–17907. 10.1074/jbc.M710609200 18408002 PMC2440607

[B15] Cai Y. Wang R. Pei F. Liang B. B. (2007). Antibacterial activity of allicin alone and in combination with β-lactams against *Staphylococcus* spp. and *Pseudomonas aeruginosa*. *J. Antibiot.* 60 335–338. 10.1038/ja.2007.45 17551215

[B16] Cavallito C. J. Bailey J. H. (1944). Allicin, the antibacterial principle of allium sativum. I. Isolation, physical properties and antibacterial action. *J. Am. Chem. Soc.* 66 1950–1951. 10.1021/ja01239a048

[B17] Couto N. Wood J. Barber J. (2016). The role of glutathione reductase and related enzymes on cellular redox homoeostasis network. *Free Radic. Biol. Med.* 95 27–42. 10.1016/j.freeradbiomed.2016.02.028 26923386

[B18] Debnath A. Ndao M. Reed S. (2013). Reprofiled drug targets ancient protozoans. *Gut Microbes* 4 66–71. 10.4161/gmic.22596 23137963 PMC3555889

[B19] Duan D. Zhang B. Yao J. Liu Y. Fang J. (2014). Shikonin targets cytosolic thioredoxin reductase to induce ROS-mediated apoptosis in human promyelocytic leukemia HL-60 cells. *Free Radic. Biol. Med.* 70 182–193. 10.1016/j.freeradbiomed.2014.02.016 24583460

[B20] Dwivedi V. P. Bhattacharya D. Singh M. Bhaskar A. Kumar S. Fatima S. (2019). Allicin enhances antimicrobial activity of macrophages during *Mycobacterium tuberculosis* infection. *J. Ethnopharmacol.* 243:111634. 10.1016/j.jep.2018.12.008 30537531

[B21] Eagle H. (1948). A paradoxical zone phenomenon in the bactericidal action of penicillin in vitro. *Science* 107 44–45. 17778247 10.1126/science.107.2767.44

[B22] Feldberg R. S. Chang S. C. Kotik A. N. Nadler M. Neuwirth Z. Sundstrom D. C. (1988). In vitro mechanism of inhibition of bacterial cell growth by allicin. *Antimicrob. Agents Chemother.* 32 1763–1768. 10.1128/AAC.32.12.1763 2469386 PMC176014

[B23] Felson D. Anderson J. Meenan R. (1990). The comparative efficacy and toxicity of second-line drugs in rheumatoid. *Arthritis Rheum.* 33 1449–1461. 1977391 10.1002/art.1780331001

[B24] Finkelstein A. E. Walz D. T. Batista V. Mizraji M. Roisman F. Misher A. (1976). Auranofin. New oral gold compound for treatment of rheumatoid arthritis. *Ann. Rheum. Dis.* 35 251–257. 10.1136/ard.35.3.251 791161 PMC1006549

[B25] French G. L. (2006). Bactericidal agents in the treatment of MRSA infections - The potential role of daptomycin. *J. Antimicrob. Chemother.* 58 1107–1117. 10.1093/jac/dkl393 17040922

[B26] Fuchs B. B. Rajamuthiah R. Souza A. C. R. Eatemadpor S. Rossoni R. D. Santos D. A. (2016). Inhibition of bacterial and fungal pathogens by the orphaned drug auranofin. *Future Med. Chem.* 8 117–132. 26808006 10.4155/fmc.15.182 PMC4976847

[B27] Gromer S. Urig S. Becker K. (2004). The thioredoxin system - from science to clinic. *Med. Res. Rev.* 24 40–89. 10.1002/med.10051 14595672

[B28] Gustafsson T. N. Osman H. Werngren J. Hoffner S. Engman L. Holmgren A. (2016). Ebselen and analogs as inhibitors of Bacillus anthracis thioredoxin reductase and bactericidal antibacterials targeting Bacillus species, *Staphylococcus aureus* and *Mycobacterium tuberculosis*. *Biochim. Biophys. Acta Gen. Subj.* 1860 1265–1271. 10.1016/j.bbagen.2016.03.013 26971857

[B29] Harbut M. B. Vilchèze C. Luo X. Hensler M. E. Guo H. Yang B. (2015). Auranofin exerts broad-spectrum bactericidal activities by targeting thiol-redox homeostasis. *Proc. Natl. Acad. Sci. U.S.A.* 112 4453–4458. 10.1073/pnas.1504022112 25831516 PMC4394260

[B30] Hu Y. Palmer S. Munoz H. Bullard J. (2014). High throughput screen identified natural product inhibitor of phenylalanyl-tRNA synthetase from *Pseudomonas aeruginosa* and *Streptococcus pneumoniae*. *Curr. Drug Discov. Technol.* 11 279–292. 25601215 10.2174/1570163812666150120154701 PMC4439361

[B31] Ianiri G. Idnrum A. L. (2015). Essential gene discovery in the Basidiomycete Cryptococcus neoformans for antifungal drug target prioritization. *mBio* 6:e02334-14. 10.1128/mBio.02334-14 25827419 PMC4453551

[B32] Ilari A. Baiocco P. Messori L. Fiorillo A. Boffi A. Gramiccia M. (2012). A gold-containing drug against parasitic polyamine metabolism: the X-ray structure of trypanothione reductase from *Leishmania infantum* in complex with auranofin reveals a dual mechanism of enzyme inhibition. *Amino Acids* 42 803–811. 10.1007/s00726-011-0997-9 21833767 PMC3266496

[B33] Jonkers D. Van Den Broek E. Van Dooren I. Thijs C. Dorant E. Hageman G. (1999). Antibacterial effect of garlic and omeprazole on *Helicobacter pylori*. *J. Antimicrob. Chemother.* 43 837–839. 10.1093/jac/43.6.837 10404325

[B34] Karlenius T. C. Tonissen K. F. (2010). Thioredoxin and cancer: a role for thioredoxin in all states of tumor oxygenation. *Cancers* 2 209–232. 10.3390/cancers2020209 24281068 PMC3835076

[B35] Katzianer D. Yano T. Rubin H. Zhu J. (2014). A high-throughput small-molecule screen to identify a novel chemical inhibitor of *Clostridium* difficile. *Int. J. Antimicrob. Agents* 44 69–73. 24837414 10.1016/j.ijantimicag.2014.03.007 PMC4062579

[B36] Kean W. F. Hart L. Buchanan W. W. (1997). Auranofin. *Br. J. Rheumatol.* 36 560–572. 9189058 10.1093/rheumatology/36.5.560

[B37] Kim W. Conery A. L. Rajamuthiah R. Fuchs B. B. Ausubel F. M. Mylonakis E. (2015). Identification of an antimicrobial agent effective against methicillin-resistant *Staphylococcus aureus* persisters using a fluorescence-based screening strategy. *PLoS One* 10:e0127640. 10.1371/journal.pone.0127640 26039584 PMC4454602

[B38] King M. D. Humphrey B. J. Wang Y. F. Kourbatova E. V. Ray S. M. Blumberg H. M. (2006). Emergence of community-acquired methicillin-resistant *Staphylococcus aureus* USA 300 clone as the predominant cause of skin and soft-tissue infections. *Ann. Intern. Med.* 144 309–317. 10.7326/0003-4819-144-5-200603070-00005 16520471

[B39] Koçkar C. Oztürk M. Bavbek N. (2001). *Helicobacter pylori* eradication with beta carotene, ascorbic acid and allicin. *Acta Medica* 44 97–100. 10.14712/18059694.2019.92 11811084

[B40] Kuo H.-M. Hsia T.-C. Chuang Y.-C. Lu H.-F. Lin S.-Y. Chung J.-G. (2004). Shikonin inhibits the growth and N-acetylation of 2-aminofluorene in *Helicobacter pylori* from ulcer patients. *Anticancer Res.* 24 1587–1592. 15274326

[B41] Lee Y. S. Lee D. Y. Kim Y. B. Lee S. W. Cha S. W. Park H. W. (2015). The mechanism underlying the antibacterial activity of shikonin against methicillin-resistant *Staphylococus aureus*. *Evid. Based Complement. Altern. Med.* 2015:520578. 10.1155/2015/520578 26265924 PMC4523682

[B42] Leng B. F. Qiu J. Z. Dai X. H. Dong J. Wang J. F. Luo M. J. (2011). Allicin reduces the production of α-toxin by *Staphylococcus aureus*. *Molecules* 16 7958–7968. 10.3390/molecules16097958 21921868 PMC6264299

[B43] Lin K. O’Brien K. M. Trujillo C. Wang R. Wallach J. B. Schnappinger D. (2016). *Mycobacterium tuberculosis* Thioredoxin Reductase is rssential for thiol redox homeostasis but plays a minor role in antioxidant defense. *PLoS Pathog.* 12:e1005675. 10.1371/journal.ppat.1005675 27249779 PMC4889078

[B44] Lu J. Holmgren A. (2014). The thioredoxin antioxidant system. *Free Radic. Biol. Med.* 66 75–87. 10.1016/j.freeradbiomed.2013.07.036 23899494

[B45] Lu J. Vlamis-Gardikas A. Kandasamy K. Zhao R. Gustafsson T. N. Engstrand L. (2013). Inhibition of bacterial thioredoxin reductase: an antibiotic mechanism targeting bacteria lacking glutathione. *FASEB J.* 27 1394–1403. 10.1096/fj.12-223305 23248236

[B46] Missall T. A. Lodge J. K. (2005). Thioredoxin reductase is essential for viability in the fungal pathogen *Cryptococcus neoformans*. *Eukaryot. Cell* 4 487–489. 10.1128/EC.4.2.487-489.2005 15701811 PMC549343

[B47] Mohammad H. Abutaleb N. S. Seleem M. N. (2020). Auranofin rapidly eradicates methicillin-resistant *Staphylococcus aureus* (MRSA) in an infected pressure ulcer mouse model. *Sci. Rep.* 10:7251. 10.1038/s41598-020-64352-2 32350417 PMC7190694

[B48] Mohammadi F. Soltani A. Ghahremanloo A. Javid H. Hashemy S. I. (2019). The thioredoxin system and cancer therapy: a review. *Cancer Chemother. Pharmacol.* 84 925–935. 10.1007/s00280-019-03912-4 31367788

[B49] Moy T. I Ball A. R. Anklesaria Z. Casadei G. Lewis K. Ausubel F. M. (2006). Identification of novel antimicrobials using a live-animal infection model. *Proc. Natl. Acad. Sci. U.S.A.* 103 10414–10419. 10.1073/pnas.0604055103 16801562 PMC1482800

[B50] Müller A. Cadenas E. Graf P. Sies H. (1984). A novel biologically active seleno-organic compound-I. Glutathione peroxidase-like activity in vitro and antioxidant capacity of PZ 51 (Ebselen). *Biochem. Pharmacol.* 33 3235–3239. 10.1016/0006-2952(84)90083-2 6487370

[B51] Muller A. Eller J. Albrecht F. Prochnow P. Kuhlmann K. Bandow J. E. (2016). Allicin induces thiol stress in bacteria through S -allylmercapto modification of protein cysteines. *J. Biol. Chem.* 291 11477–11490. 10.1074/jbc.M115.702308 27008862 PMC4882420

[B52] Nemeth J. Oesch G. Kuster S. P. (2015). Bacteriostatic versus bactericidal antibiotics for patients with serious bacterial infections: systematic review and meta-analysis. *J. Antimicrob. Chemother.* 70 382–395. 10.1093/jac/dku379 25266070

[B53] O’Gara E. Hill D. Maslin D. (2000). Activities of garlic oil, garlic powder, and their diallyl constituents against *Helicobacter pylori*. *Appl. Environ. Microbiol.* 66 2269–2273. 10788416 10.1128/aem.66.5.2269-2273.2000 PMC101489

[B54] Ollinger J. Kumar A. Roberts D. Bailey M. Casey A. Parish T. (2019). A high-throughput whole cell screen to identify inhibitors of *Mycobacterium tuberculosis*. *PLoS One* 14:e0205479. 10.1371/journal.pone.0205479 30650074 PMC6334966

[B55] Owings J. P. McNair N. N. Mui Y. F. Gustafsson T. N. Holmgren A. Contel M. (2016). Auranofin and N-heterocyclic carbene gold-analogs are potent inhibitors of the bacteria *Helicobacter pylori*. *FEMS Microbiol. Lett.* 363 1–6. 10.1093/femsle/fnw148 27279627

[B56] Pankey G. A. Sabath L. D. (2004). Clinical relevance of bacteriostatic versus bactericidal mechanisms of action in the treatment of Gram-positive bacterial infections. *Clin. Infect. Dis.* 38 864–870. 10.1086/381972 14999632

[B57] Parnham M. Sies H. (2000). Ebselen: prospective therapy for cerebral ischaemia. *Expert Opin. Investig. Drugs* 9 607–619. 10.1517/13543784.9.3.607 11060699

[B58] Parsonage D. Sheng F. Hirata K. Debnath A. Mckerrow J. H. Reed S. L. (2016). X-ray structures of thioredoxin and thioredoxin reductase from Entamoeba histolytica and prevailing hypothesis of the mechanism of auranofin action. *J. Struct. Biol.* 194 180–190. 10.1016/j.jsb.2016.02.015.X-ray 26876147 PMC5003402

[B59] Pérez-Köhler B. García-Moreno F. Bayon Y. Pascual G. Bellón J. M. (2015a). Inhibition of *Staphylococcus aureus* adhesion to the surface of a reticular heavyweight polypropylene mesh soaked in a combination of chlorhexidine and allicin: an in vitro study. *PLoS One* 10:e0126711. 10.1371/journal.pone.0126711 25962163 PMC4427482

[B60] Pérez-Köhler B. García-Moreno F. Brune T. Pascual G. Bellón J. M. (2015b). Preclinical bioassay of a polypropylene mesh for hernia repair pretreated with antibacterial solutions of chlorhexidine and allicin: an in vivo study. *PLoS One* 10:e0142768. 10.1371/journal.pone.0142768 26556805 PMC4640885

[B61] Potamitou A. Holmgren A. Vlamis-Gardikas A. (2002). Protein levels of *Escherichia coli* thioredoxins and glutaredoxins and their relation to null mutants, growth phase, and function. *J. Biol. Chem.* 277 18561–18567. 10.1074/jbc.M201225200 11893749

[B62] Prasetyoputro A. Jarrrad A. M. Cooper M. A. Blaskovich M. A. T. (2019). The Eagle effect and antibiotic-induced persistence: two sides of teh same coin? *Trends Microbiol.* 27 339–354. 30448198 10.1016/j.tim.2018.10.007

[B63] Rajamuthiah R. Fuchs B. B. Jayamani E. Kim Y. Larkins-Ford J. Conery A. (2014). Whole animal automated platform for drug discovery against multi-drug resistant *Staphylococcus aureus*. *PLoS One* 9:e89189. 10.1371/journal.pone.0089189 24586584 PMC3929655

[B64] Schewe T. (1995). Molecular actions of ebselen-an antiinflammatory antioxidant. *Gen. Pharmacol.* 26 1153–1169. 10.1016/0306-3623(95)00003-J 7590103

[B65] St. John G. Brot N. Ruan J. Erdjument-Bromage H. Tempst P. Weissbach H. (2001). Peptide methionine sulfoxide reductase from *Escherichia coli* and *Mycobacterium tuberculosis* protects bacteria against oxidative damage from reactive nitrogen intermediates. *Proc. Natl. Acad. Sci. U.S.A.* 98 9901–9906. 10.1073/pnas.161295398 11481433 PMC55550

[B66] Thangamani S. Mohammad H. Abushahba M. F. N. Sobreira T. J. P. Hedrick V. E. Paul L. N. (2016a). Antibacterial activity and mechanism of action of auranofin against multi-drug resistant bacterial pathogens. *Sci. Rep.* 6:22571. 10.1038/srep22571 26936660 PMC4776257

[B67] Thangamani S. Mohammad H. Abushahba M. F. N. Sobreira T. J. P. Seleem M. N. (2016b). Repurposing auranofin for the treatment of cutaneous staphylococcal infections. *Int. J. Antimicrob. Agents* 47 195–201. 10.1016/j.ijantimicag.2015.12.016 26895605 PMC4792765

[B68] Thangamani S. Younis W. Seleem M. N. (2015a). Repurposing clinical molecule ebselen to combat drug resistant pathogens. *PLoS One* 10:e0133877. 10.1371/journal.pone.0133877 26222252 PMC4519285

[B69] Thangamani S. Younis W. Seleem M. N. (2015b). Repurposing ebselen for treatment of multidrug-resistant staphylococcal infections. *Sci. Rep.* 5:11596. 10.1038/srep11596 26111644 PMC4481386

[B70] Tharmalingam N. Ribeiro N. da Silva D. Naik M. Cruz L. Kim W. (2019). Auranofin is an effective agent against clinical isolates of *Staphylococcus aureus*. *Future Med. Chem.* 11 1417–1425. 31298580 10.4155/fmc-2018-0544 PMC7202268

[B71] Torres N. S. Abercrombie J. J. Srinivasan A. Lopez-Ribot J. L. Ramasubramanian A. K. Leung K. P. (2016). Screening a commercial library of pharmacologically active small molecules against *Staphylococcus aureus* biofilms. *Antimicrob. Agents Chemother.* 60 5663–5672. 10.1128/AAC.00377-16 27401577 PMC5038268

[B72] Trivedi A. Singh N. Bhat S. A. Gupta P. Kumar A. (2012). Redox biology of tuberculosis pathogenesis. *Adv. Microb. Physiol.* 60 263–324. 10.1016/B978-0-12-398264-3.00004-8 22633061

[B73] Uziel O. Borovok I. Schreiber R. Cohen G. Aharonowitz Y. (2004). Transcriptional regulation of the *Staphylococcus aureus* thioredoxin and thioredoxin reductase genes in response to oxygen and disulfide stress. *J. Bacteriol.* 186 326–334. 10.1128/JB.186.2.326 14702300 PMC305758

[B74] Vegara S. Funes L. Martí N. Saura D. Micol V. Valero M. (2011). Bactericidal activities against pathogenic bacteria by selected constituents of plant extracts in carrot broth. *Food Chem.* 128 872–873. 10.1016/j.foodchem.2011.03.109

[B75] Wald-Dickler N. Holtom P. Spellberg B. (2018). Busting the myth of “static vs cidal”: a systemic literature review. *Clin. Infect. Dis.* 66 1470–1474. 10.1093/cid/cix1127 29293890 PMC5905615

[B76] Williams C. H. Arscott L. Muller S. Lennon B. (2000). Thioredoxin reductase: two modes of catalysis have evolved. *Eur. J. Biochem.* 267 6110–6117. 11012662 10.1046/j.1432-1327.2000.01702.x

[B77] Windle H. J. Fox Á Eidhin D. N. Kelleher D. (2000). The thioredoxin system of *Helicobacter pylori*. *J. Biol. Chem.* 275 5081–5089. 10.1074/jbc.275.7.5081 10671551

[B78] Zhai H. Pan J. Pang E. Bai B. (2014). Lavage with allicin in combination with vancomycin inhibits biofilm formation by *Staphylococcus epidermidis* in a rabbit model of prosthetic joint infection. *PLoS One* 9:e102760. 10.1371/journal.pone.0102760 25025650 PMC4099135

[B79] Zheng C. Guo S. Tennant W. G. Pradhan P. K. Black K. A. Dos Santos P. C. (2019). The thioredoxin system reduces protein persulfide intermediates formed during the synthesis of thio-cofactors in *Bacillus subtilis*. *Biochemistry* 58 1892–1904. 10.1021/acs.biochem.9b00045 30855939

